# Supplementary figures and images for: A curriculum learning approach to training antibody language models
Source: PLoS Comput Biol. 2025 Sep 11;21(9):e1013473. doi: 10.1371/journal.pcbi.1013473 (PMC12468933; doi:10.1371/journal.pcbi.1013473)

**A** Paired Model  
(Chains Reversed)

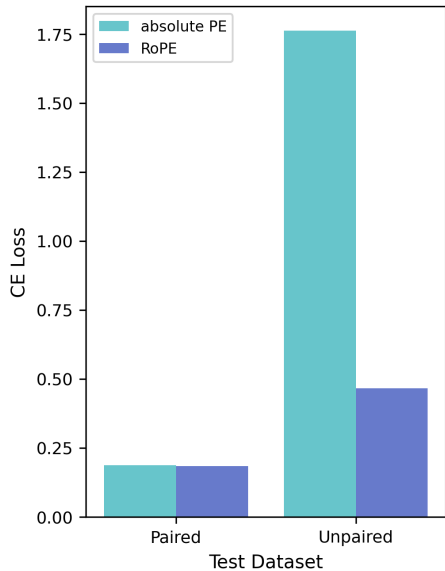

**B**

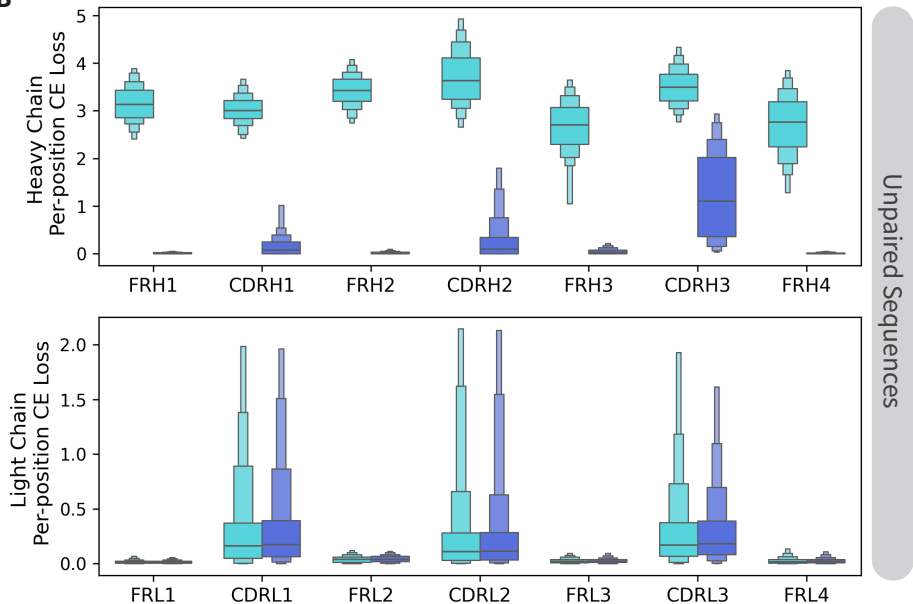

Supplement: S1 Fig — (A) CE loss on paired and unpaired test datasets of ~10k sequences each. (B) Per-position CE loss of models on 1k sequences from the unpaired dataset. (PDF) [file pcbi.1013473.s001.pdf]
