# Supplementary material for: A curriculum learning approach to training antibody language models
Source: PLoS Comput Biol. 2025 Sep 11;21(9):e1013473. doi: 10.1371/journal.pcbi.1013473 (PMC12468933; doi:10.1371/journal.pcbi.1013473)
Supplement: S1 Table — Mixed, paired-only, and unpaired-only models were trained with 5 different separators. Separators were placed between chains in paired sequences and unpaired sequences based on the chain (end of the heavy chains and the beginning of the light chains). Models were assessed on paired and unpaired test datasets, each containing ~ 10k sequences. (PDF) [file pcbi.1013473.s002.pdf]

Separator	*Mixed Models*				*Paired Models*		*Unpaired Models*	
	Paired Data		Unpaired Data					
	CE Loss	Accuracy	CE Loss	Accuracy	CE Loss	Accuracy	CE Loss	Accuracy
None	0.1835	**0.9510**	0.3571	**0.9133**	0.1856	**0.9507**	0.3404	0.9177
<cls>	**0.1828**	<u>0.9509</u>	**0.3561**	**0.9133**	**0.1851**	<u>0.9506</u>	<u>0.3389</u>	<u>0.9179</u>
<sep>	<u>0.1831</u>	<u>0.9509</u>	<u>0.3565</u>	<u>0.9131</u>	<u>0.1852</u>	<u>0.9506</u>	**0.3386**	**0.9180**
<cls><cls>	0.1835	0.9508	0.3601	0.9125	0.1854	<u>0.9506</u>	0.3423	0.9175
<sep><sep>	0.1833	0.9508	0.3604	0.9124	0.1856	0.9505	0.3427	0.9174
