# Supplementary material for: A curriculum learning approach to training antibody language models
Source: PLoS Comput Biol. 2025 Sep 11;21(9):e1013473. doi: 10.1371/journal.pcbi.1013473 (PMC12468933; doi:10.1371/journal.pcbi.1013473)
Supplement: S2 Table — Mixed models were trained with increasing percentages of unpaired data. Models were assessed on paired and unpaired test datasets, each containing ~ 10k sequences. (PDF) [file pcbi.1013473.s003.pdf]

	Paired		*Unpaired*	
**Unpaired %**	**CE Loss**	**Accuracy**	**CE Loss**	**Accuracy**
37.5	**0.1831**	0.9508	0.3635	0.9111
50	**0.1831**	**0.9509**	0.3565	0.9131
62.5	0.1833	**0.9509**	0.3508	0.9148
75	0.1839	0.9508	**0.3463**	**0.9164**
