# Supplementary material for: A curriculum learning approach to training antibody language models
Source: PLoS Comput Biol. 2025 Sep 11;21(9):e1013473. doi: 10.1371/journal.pcbi.1013473 (PMC12468933; doi:10.1371/journal.pcbi.1013473)
Supplement: S2 Fig — (A) Unpaired probability curves for the five pilot models. (B) CE loss on paired and unpaired test datasets of ~10k sequences. (C) Mixed models accuracy at predicting CDRH3 of 1k paired sequences from the test set. Results for classification tasks, which are one (D) Native vs Random pair classification, two Healthy Donor vs Flu vs CoV specificity classifications using (E) paired and (F) unpaired sequences, and two Healthy Donor vs CoV specificity classifications using (H) paired and (I) unpaired sequences. (G) Pair classification results were split into mutated, unmutated, and mismatched pairs. Metrics on classification tasks are mean and standard error, with the highest values bolded and the second highest values underlined. (PDF) [file pcbi.1013473.s004.pdf]

A

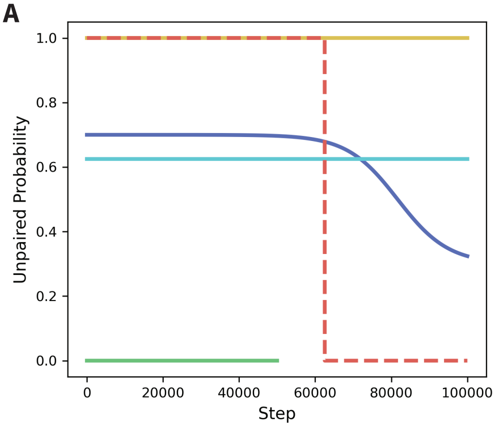

B

Model	Paired CE Loss	Paired Accuracy	Unpaired CE Loss	Unpaired Accuracy
unpaired	0.3177	0.9275	**0.3391**	**0.9180**
constant	**0.1833**	**0.9509**	0.3508	0.9148
curriculum	**0.1833**	**0.9509**	0.3501	0.9152
finetuned	0.1834	**0.9509**	0.4476	0.9027
paired	0.1852	0.9506	0.4720	0.9004

constant
curriculum
finetuned
unpaired
paired

C

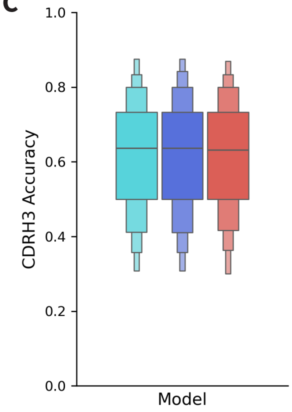

D

Classification	Model	Accuracy	F1	MCC	AUC	AUPR
Native vs Random Pairs	unpaired	0.5174 (± 0.0014)	**0.5320** (± 0.0042)	0.0349 (± 0.0028)	0.5255 (± 0.0023)	0.5207 (± 0.0030)
	constant	0.6022 (± 0.0008)	0.4762 (± 0.0020)	0.2331 (± 0.0020)	0.6361 (± 0.0020)	0.6532 (± 0.0026)
	curriculum	0.6073 (± 0.0018)	0.5013 (± 0.0030)	0.2370 (± 0.0039)	0.6437 (± 0.0031)	0.6615 (± 0.0029)
	finetuned	0.5872 (± 0.0017)	0.5021 (± 0.0034)	0.1857 (± 0.0036)	0.6209 (± 0.0018)	0.6394 (± 0.0028)
	paired	**0.6243** (± 0.0013)	0.5066 (± 0.0034)	**0.2829** (± 0.0021)	**0.6665** (± 0.0027)	**0.6837** (± 0.0029)

E / F

Classification	Model	Accuracy	F1	MCC
Paired HD vs Flu vs CoV	unpaired	0.5450 (± 0.0025)	0.5402 (± 0.0024)	0.3305 (± 0.0051)
	constant	0.5728 (± 0.0038)	0.5725 (± 0.0036)	0.3660 (± 0.0062)
	curriculum	0.5903 (± 0.0056)	0.5923 (± 0.0054)	0.3879 (± 0.0080)
	finetuned	0.6132 (± 0.0018)	0.6136 (± 0.0017)	0.4213 (± 0.0027)
	paired	**0.6155** (± 0.0041)	**0.6170** (± 0.0041)	**0.4243** (± 0.0061)
Unpaired HD vs Flu vs CoV	unpaired	0.5693 (± 0.0086)	0.5704 (± 0.0087)	0.3580 (± 0.0136)
	constant	0.5794 (± 0.0026)	0.5809 (± 0.0025)	0.3742 (± 0.0046)
	curriculum	0.5730 (± 0.0045)	0.5751 (± 0.0045)	0.3628 (± 0.0066)
	finetuned	0.5975 (± 0.0037)	0.5985 (± 0.0036)	0.3975 (± 0.0055)
	paired	**0.6098** (± 0.0032)	**0.6110** (± 0.0031)	**0.4157** (± 0.0050)

G

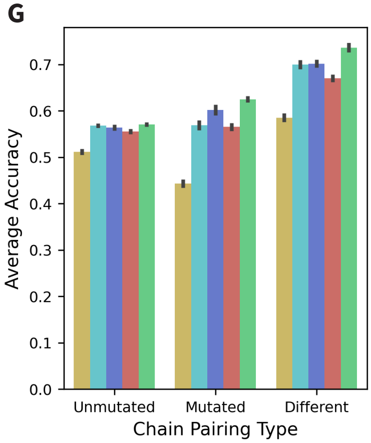

H / I

Classification	Model	Accuracy	F1	MCC	AUC	AUPR
Paired HD vs CoV	unpaired	0.6555 (± 0.0020)	0.7078 (± 0.0017)	0.3331 (± 0.0042)	0.7443 (± 0.0015)	0.7410 (± 0.0019)
	constant	0.7083 (± 0.0014)	0.7169 (± 0.0028)	0.4175 (± 0.0031)	0.7795 (± 0.0020)	0.7751 (± 0.0029)
	curriculum	0.7029 (± 0.0018)	0.7067 (± 0.0020)	0.4060 (± 0.0035)	0.7727 (± 0.0019)	0.7679 (± 0.0033)
	finetuned	0.7107 (± 0.0010)	0.7137 (± 0.0006)	0.4216 (± 0.0019)	0.7830 (± 0.0004)	0.7799 (± 0.0013)
	paired	**0.7140** (± 0.0016)	**0.7127** (± 0.0019)	**0.4281** (± 0.0031)	**0.7855** (± 0.0005)	**0.7813** (± 0.0014)
Unpaired HD vs CoV	unpaired	0.6870 (± 0.0023)	0.7047 (± 0.0029)	0.3767 (± 0.0049)	0.7563 (± 0.0016)	0.7550 (± 0.0022)
	constant	0.6917 (± 0.0027)	**0.7081** (± 0.0031)	0.3860 (± 0.0056)	0.7610 (± 0.0016)	0.7554 (± 0.0016)
	curriculum	0.6901 (± 0.0024)	0.7044 (± 0.0030)	0.3820 (± 0.0051)	0.7577 (± 0.0027)	0.7547 (± 0.0032)
	finetuned	0.6958 (± 0.0027)	0.7042 (± 0.0029)	0.3923 (± 0.0054)	0.7638 (± 0.0008)	0.7590 (± 0.0013)
	paired	**0.7022** (± 0.0025)	0.7026 (± 0.0029)	**0.4044** (± 0.0050)	**0.7685** (± 0.0013)	**0.7635** (± 0.0011)
