# Supplementary material for: A curriculum learning approach to training antibody language models
Source: PLoS Comput Biol. 2025 Sep 11;21(9):e1013473. doi: 10.1371/journal.pcbi.1013473 (PMC12468933; doi:10.1371/journal.pcbi.1013473)
Supplement: S3 Fig — Per-position CE loss on 1k memory B-cell (A) paired and (B) unpaired sequences. Results for Healthy Donor vs CoV specificity classification tasks with (C) paired and (D) unpaired sequences. Metrics on classification tasks are mean and standard error, with the highest values bolded and the second highest values underlined. (PDF) [file pcbi.1013473.s006.pdf]

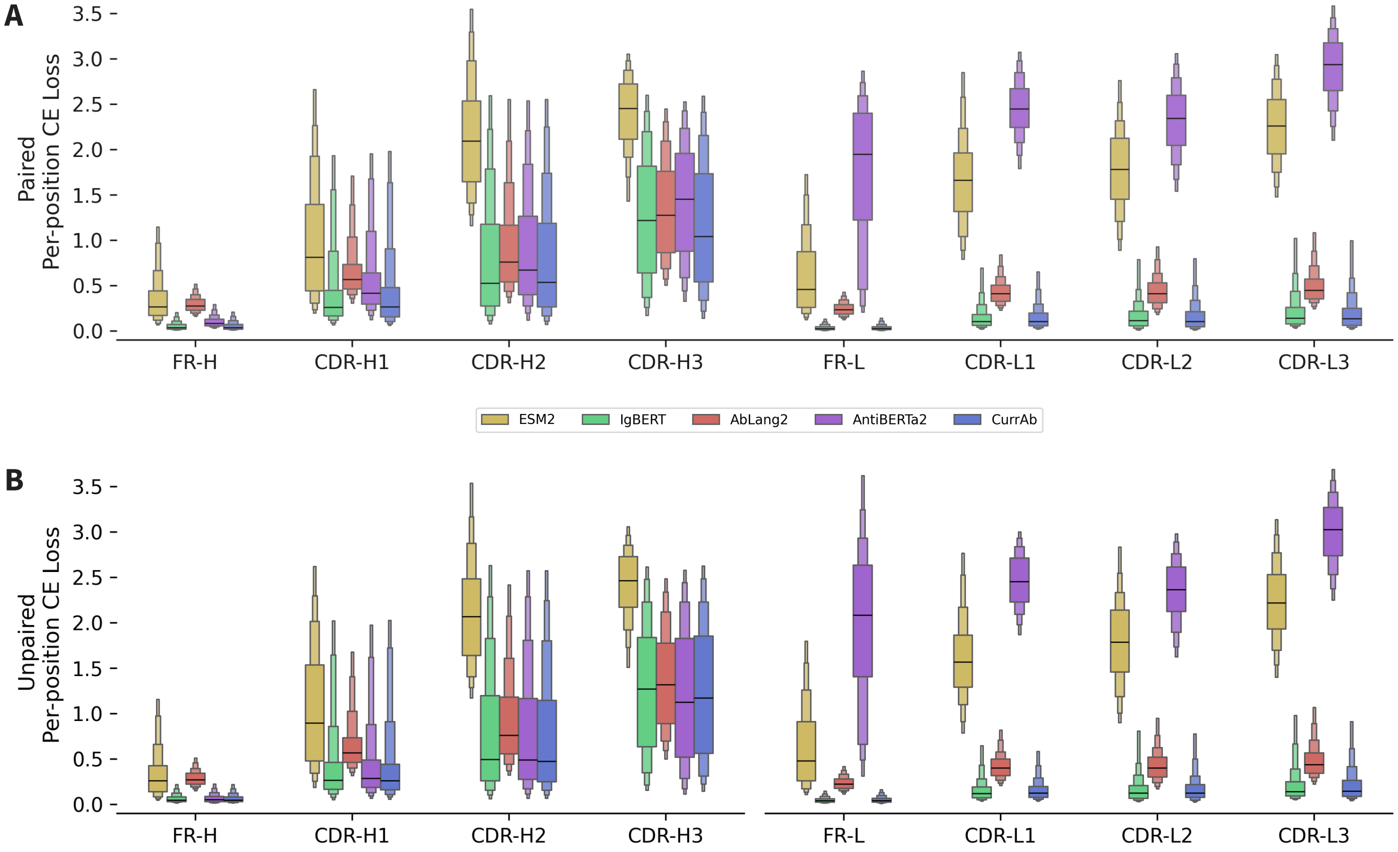

C

Classification	Model	Accuracy	F1	AUC	AUPR	MCC
Paired HD vs CoV	ESM2	0.6799 (± 0.0011)	0.6832 (± 0.0014)	0.7423 (± 0.0010)	0.7332 (± 0.0024)	0.3599 (± 0.0022)
	IgBERT	0.6756 (± 0.0028)	0.6733 (± 0.0040)	0.7344 (± 0.0023)	0.7045 (± 0.0036)	0.3513 (± 0.0055)
	IgT5	0.6763 (± 0.0038)	0.6822 (± 0.0042)	0.7333 (± 0.0044)	0.7144 (± 0.0059)	0.3535 (± 0.0074)
	AbLang2	0.7187 (± 0.0033)	0.7237 (± 0.0027)	0.7943 (± 0.0012)	0.7764 (± 0.0016)	0.4377 (± 0.0065)
	AntiBERTa2	0.6913 (± 0.0019)	0.6982 (± 0.0019)	0.7639 (± 0.0010)	0.7642 (± 0.0011)	0.3831 (± 0.0037)
	CurrAb	**0.7626** (± 0.0023)	**0.7669** (± 0.0016)	**0.8452** (± 0.0015)	**0.8381** (± 0.0023)	**0.5255** (± 0.0045)

D

Classification	Model	Accuracy	F1	AUC	AUPR	MCC
Unpaired HD vs CoV	ESM2	0.6599 (± 0.0018)	0.6586 (± 0.0015)	0.7162 (± 0.0022)	0.7071 (± 0.003)	0.3198 (± 0.0035)
	IgBERT	0.6613 (± 0.0025)	0.6559 (± 0.0043)	0.7189 (± 0.0014)	0.7015 (± 0.0029)	0.3229 (± 0.0049)
	IgT5	0.6583 (± 0.0040)	0.6861 (± 0.0037)	0.7170 (± 0.0035)	0.6886 (± 0.0034)	0.3219 (± 0.0078)
	AbLang2	0.7081 (± 0.0016)	0.7140 (± 0.0021)	0.7785 (± 0.0015)	0.7574 (± 0.0013)	0.4167 (± 0.0033)
	AntiBERTa2	0.7205 (± 0.0018)	0.7193 (± 0.0027)	0.7927 (± 0.0021)	0.7893 (± 0.0018)	0.4410 (± 0.0035)
	CurrAb	**0.7218** (± 0.0014)	**0.7316** (± 0.0012)	**0.8011** (± 0.0010)	**0.7992** (± 0.0014)	**0.4448** (± 0.0027)
