# Supplementary material for: A curriculum learning approach to training antibody language models
Source: PLoS Comput Biol. 2025 Sep 11;21(9):e1013473. doi: 10.1371/journal.pcbi.1013473 (PMC12468933; doi:10.1371/journal.pcbi.1013473)
Supplement: S4 Table — Corresponding figure, model name, and equation values (A, B, shift, and k) are listed for each curriculum model. (PDF) [file pcbi.1013473.s007.pdf]

Figure	Model	A	B	Shift	k
3A-B	max1	1	1	0.6250	20
	max0.9	0.8	0.9	0.6563	20
	max0.8	0.6	0.8	0.7085	20
	max0.7	0.4	0.7	0.8137	20
3C-D	k = 10	0.4	0.7	0.8291	10
	k = 15	0.4	0.7	0.8166	15
	k = 20	0.4	0.7	0.8137	20
	k = 50	0.4	0.7	0.8125	50
3E-F	all	0.4	0.7	0.8137	20
4, 5	CurrAb	0.4	0.7	0.8166	15
